# How low can you go? Examining the effects of brief online training and post-training consultation dose on implementation mechanisms and outcomes for measurement-based care

**DOI:** 10.1186/s43058-022-00325-y

**Published:** 2022-07-22

**Authors:** Aaron R. Lyon, Freda F. Liu, Elizabeth H. Connors, Kevin M. King, Jessica I. Coifman, Heather Cook, Erin McRee, Kristy Ludwig, Amy Law, Shannon Dorsey, Elizabeth McCauley

**Affiliations:** 1 Department of Psychiatry and Behavioral Sciences, University of Washington, 6200 NE 74th Street, Suite 100, Seattle, WA 98115 USA; 2 Department of Psychiatry, Yale University, 389 Whitney Avenue, Office 106, New Haven, CT 06511 USA; 3 Department of Psychology, University of Washington, Guthrie Hall, Box 351525, Seattle, WA 98195 USA; 4 Graduate Medical Education, University of Washington, Learning Gateway, Box 358220, Seattle, WA 98109 USA

**Keywords:** Implementation strategy optimization, Measurement-based care, Education sector mental health, Mental health treatment in schools, Consultation

## Abstract

**Background:**

Initial training and ongoing post-training consultation (i.e., ongoing support following training, provided by an expert) are among the most common implementation strategies used to change clinician practice. However, extant research has not experimentally investigated the optimal dosages of consultation necessary to produce desired outcomes. Moreover, the degree to which training and consultation engage theoretical implementation mechanisms—such as provider knowledge, skills, and attitudes—is not well understood. This study examined the effects of a brief online training and varying dosages of post-training consultation (BOLT+PTC) on implementation mechanisms and outcomes for measurement-based care (MBC) practices delivered in the context of education sector mental health services.

**Methods:**

A national sample of 75 clinicians who provide mental health interventions to children and adolescents in schools were randomly assigned to BOLT+PTC or control (services as usual). Those in BOLT+PTC were further randomized to 2-, 4-, or 8-week consultation conditions. Self-reported MBC knowledge, skills, attitudes, and use (including standardized assessment, individualized assessment, and assessment-informed treatment modification) were collected for 32 weeks. Multilevel models were used to examine main effects of BOLT+PTC versus control on MBC use at the end of consultation and over time, as well as comparisons among PTC dosage conditions and theorized mechanisms (skills, attitudes, knowledge).

**Results:**

There was a significant linear effect of BOLT+PTC over time on standardized assessment use (*b* = .02, *p* < .01), and a significant quadratic effect of BOLT+PTC over time on individualized assessment use (*b* = .04, *p* < .001), but no significant effect on treatment modification. BOLT + any level of PTC resulted in higher MBC knowledge and larger growth in MBC skill over the intervention period as compared to control. PTC dosage levels were inconsistently predictive of outcomes, providing no clear evidence for added benefit of higher PTC dosage.

**Conclusions:**

Online training and consultation in MBC had effects on standardized and individualized assessment use among clinicians as compared to services as usual with no consistent benefit detected for increased consultation dosage. Continued research investigating optimal dosages and mechanisms of these established implementation strategies is needed to ensure training and consultation resources are deployed efficiently to impact clinician practices.

**Trial registration:**

ClinicalTrials.gov NCT05041517. Retrospectively registered on 10 September 2021.

**Supplementary Information:**

The online version contains supplementary material available at 10.1186/s43058-022-00325-y.

Contributions to the literature

- Initial training and post-training consultation are cornerstone implementation strategies, but their mechanisms of action are rarely explicitly assessed, and little is known about the effects of consultation dosage.
- This study is innovative in that it evaluated implementation mechanisms for training and consultation to support the use of measurement-based care in education sector mental health services, and is the first to experimentally manipulate consultation dosage.
- Results suggest that the implementation strategies were effective in changing some MBC practices among clinicians (i.e., use of standardized and individualized assessment), but little evidence was found for a consistent benefit of higher dosages of consultation. This may indicate new, unexplored efficiencies surrounding the implementation of evidence-based practices in mental health.

## Implementation strategies: training and consultation

Over the past two decades, studies have repeatedly explored the extent to which initial training must be supplemented with additional implementation supports to effect meaningful changes in professional practice. Many reviews indicate that post-training consultation (i.e., ongoing support following training, provided by an expert with the goal of improving implementation and practice of an EBP) [1] or other supports are necessary, but the strength of the evidence remains mixed [2–5]. Recent literature points to the limitations of “workshop only” trainings and suggests that they are likely to have a greater impact on the adherence and competence of practitioners when augmented with practice-specific implementation supports and/or ongoing consultation to promote workplace-specific adaptations and skill application [6–8]. As such, initial training and post-training consultation are two cornerstone—and complementary—implementation strategies to support the implementation of evidence-based practices [9, 10]. This is especially true in mental health care, where most evidence-based practices are complex psychosocial processes that require significant professional development support to implement effectively [11].

Among the numerous implementation strategies that have been identified to potentially promote adoption, delivery, and sustainment of interventions [12, 13], initial training and post-training consultation have often proven effective for evidence-based mental health practice implementation [4, 9, 10]. Initial training generally refers to a combination of didactic content covering intervention materials or protocols and active or experiential learning such as in vivo demonstration or role plays to apply skills and receive feedback [2]. Ongoing, post-training consultation refers to practice-specific coaching by one or more experts in the intervention strategy [14]. In the context of mental health service delivery, consultation is different from supervision in that the consultant may be internal or external to the organization and does not have direct authority over the implementer [15]. Moreover, supervision can be administrative in nature [16] and consultation specifically refers to case-specific skill application and refinement of an evidence-based clinical practice [17, 18]. Together, initial training and post-training consultation are complementary and foundational implementation strategies.

Training is the starting point for many professional behavior change efforts and is associated with initial, post-training improvements in provider attitudes, knowledge, and skill acquisition [5]. Increasingly, training for mental health clinicians is provided online, something that has only intensified following the emergence of the COVID-19 pandemic [19]. Fortunately, online training is at least as effective as in-person approaches [20–23], eliminates training-related travel time, reduces costs, and allows for self-paced administration of content, thus improving accessibility and feasibility [21, 24].

Importantly, initial training likely necessitates the addition of post-training consultation or support to effect changes in professional practice [2–5, 25, 26]. However, the optimal duration or frequency of ongoing supports following initial training is not well known. In fact, the most recent review on this topic found that post-training consultation was variably effective in improving uptake [5]. Inconsistencies in these findings suggest that it is important to better understand the processes through which consultation improves implementation outcomes.

### Consultation dose

Although post-training consultation is a promising implementation strategy, very little is known about the optimal frequency and duration (i.e., dose) of post-training consultation to ensure its impact. In fact, a recent review of randomized controlled trials concluded that variations in consultation dose across studies precluded their ability to conclusively identify the effect of consultation practices [5]. Efficiency is important given that post-training consultation can easily increase the cost of training by 50% or more [27]. An uncontrolled study found that higher consultation doses were associated with greater changes in knowledge and attitudes surrounding an evidence-based intervention protocol for youth anxiety [28]. Although the moderating effects of consultation dose are typically examined observationally or quasi-experimentally [24], the current study explores consultation dose by manipulating it experimentally.

## Implementation mechanisms: provider knowledge, skill, and attitudes

Despite increasingly precise identification of implementation strategies [13], there is little information available about *how* implementation strategies exert their influence. To ensure implementation strategies accurately match barriers and promote parsimonious and efficient change efforts, current research has focused on the identification of *implementation mechanisms* [29]. Implementation mechanisms are the processes or events through which an implementation strategy operates to affect desired implementation outcomes [30]. Unfortunately, systematic reviews indicate few studies have experimentally evaluated implementation mechanisms of change [30, 31]. Whether in schools or other healthcare delivery sectors, there is growing recognition that implementation strategy optimization will be greatly facilitated by clear identification and testing of the mechanisms through which implementation outcomes are improved [29, 32, 33].

Despite the ubiquity of training and consultation in the implementation of evidence-based mental health services, few studies have articulated or evaluated their mechanisms. Theorized mechanisms for training and consultation include provider changes in (1) knowledge, (2) attitudes, and (3) acquisition of skills. Knowledge and attitudes surrounding new practices are among the most frequently identified candidate mechanisms for training efforts [34]. Indeed, previous studies have shown that training often improves EBP knowledge, and, in turn, more knowledgeable and competent clinicians have been found to exhibit superior training and consultation outcomes [34–36]. Positive attitudes are often predictive of provider adoption, engagement in training and consultation, adherence, and skill [37–40]. Further, training has been found to enhance initial skill acquisition [5, 41] and post-training consultation subsequently reinforces these gains and helps to maintain clinician skills [42]. In one study of community mental health clinicians’ acquisition of evidence-based assessment practices following training in a flexible psychotherapy protocol, attitudes improved following training alone, but skill continued to improve over the course of consultation [43]. Other studies have found similar evidence for the value of consultation for maintaining and promoting these changes [25].

## Measurement-based care in education sector mental health

The current study focuses on understanding the impact of training and consultation strategies for measurement-based care (MBC)—an evidence-based practice that involves the ongoing use of progress and outcome data to inform decision making and adjust treatment [44, 45]—on implementation mechanisms and outcomes within mental health services delivered in the education sector. MBC can be used to enhance any underlying treatment and has received extensive empirical support for its ability to improve outcomes in adult services [45, 46], with evidence rapidly accruing for children [47–50]. MBC involves both standardized (e.g., symptom rating scales with clinical norms/cutoffs) and individualized assessment measures (e.g., client-specific goals tracked quantitatively over time, such as days of school attendance) [51–53] as well as a focus on reviewing progress data with the patient to inform collaborative decisions about individualized treatment adjustments [54, 55].

MBC is particularly suited for treatment delivered in schools, where it is perceived by clinicians and students to be feasible, acceptable, and to facilitate progress toward treatment goals [56–58]. Research over the past 25 years has consistently found that schools are the most common service setting for the delivery of child and adolescent mental healthcare [59, 60]. Yet, use of evidence-based mental health treatment in school settings—including MBC—has lagged behind services in other sectors [61, 62].

The implementation of MBC in the education sector is consistent with calls to enhance the quality of services among settings, clinicians, and service recipients who would not otherwise have access to evidence-based care [63, 64]. However, while pragmatic implementation approaches are greatly needed to support MBC across service sectors, few established implementation strategies exist [54] and even fewer studies have evaluated strategies for improving MBC use among school clinicians. Existing training and consultation strategies used to implement MBC practices in schools have resulted in improvements in knowledge, attitudes, and use of practices initially and over time [43, 65], pointing to them as potential mechanisms for MBC implementation strategy effects.

## Current study

The current study was designed to better understand the impact of a brief online training (BOLT) and post-training consultation (PTC) on putative implementation mechanisms as well as MBC penetration, an indicator of the number of service recipients receiving MBC out of the total number possible [66]. This study focused on increasing providers’ use of standardized assessments, individualized assessments, and assessment-informed treatment modifications (all components of MBC practice) [45]. Moreover, our design facilitated examination of the effects of PTC dosage levels on outcomes via its experimental design. Accordingly, the current study examined three specific aims by testing (1) the impact BOLT+PTC supports on MBC practices; (2) differential effects of a 2-, 4- or 8-week dosage of consultation on MBC practices; and (3) the impact on implementation mechanisms such as MBC knowledge, skill, and attitudes (i.e., perceived benefit and perceived harm) that are hypothesized to activate favorable MBC implementation outcomes.

## Methods

### Participants

Participants included a national sample of *N* = 75 clinicians who provide mental health interventions to students on their school campus. Participants were Master’s level or above; primarily female (*n* = 68, 91%) and White (*n* = 55, 73%); and working in elementary (students approximately 5–12 years old), middle (12–14 years), and high schools (14–19 years). The most common role was mental health counselor, followed by school social worker, school psychologist, school counselor, and other professional roles. Demographic and professional characteristics for participating clinicians are presented in Table 1.

**Table 1:** Clinician participant demographic, professional, and caseload characteristics

Characteristic	Intervention group		Control group	
	***N***	%	***N***	%
Gender				
Female	32	86.5%	36	94.7%
Male	5	13.5%	2	5.3%
Latino				
Yes	4	10.8%	3	7.9%
No	33	89.2%	35	92.1%
Race				
Asian	1	2.7%	0	0.0%
Black or African-American	7	18.9%	6	15.8%
Native Hawaiian or Other Pacific Islander	1	2.7%	0	0.0%
White or Caucasian	25	67.6%	30	78.9%
Multi-racial	3	8.1%	2	5.3%
Age				
18 to 24 years old	0	0.0%	1	2.6%
25 to 34 years old	13	35.1%	10	26.3%
35 to 44 years old	13	35.1%	16	42.1%
45 to 54 years old	7	18.9%	8	21.1%
55 to 64 years old	4	10.8%	3	7.9%
Primary professional role				
School Psychologist	6	16.2%	5	13.2%
School Counselor	2	5.4%	2	5.3%
School Social Worker	9	24.3%	14	36.8%
Mental Health Counselor	15	40.5%	12	31.6%
Other	5	13.5%	5	13.2%
Years in role				
< 5 years	14	37.8%	12	31.6%
5–9 years	12	32.4%	10	26.3%
10–14 years	4	10.8%	6	15.8%
15–19 years	4	10.8%	7	18.4%
20+ years	3	8.1%	3	7.9%
Geography				
Midwest	10	27.0%	17	44.7%
Northeast	10	27.0%	6	15.8%
Southeast	12	32.4%	13	34.2%
Southwest	0	0.0%	0	0.0%
West	5	13.5%	2	5.3%
Urbanicity				
Rural	5	13.5%	9	23.7%
Suburban	12	32.4%	9	23.7%
Urban	12	32.4%	14	36.8%
Mixed	7	18.9%	6	15.8%
Other	1	2.7%	0	0.0%
Caseload size (# students seen/week)				
5 or fewer	1	2.7%	3	7.9%
6 - 9	5	13.5%	8	21.1%
10 - 14	8	21.6%	6	15.8%
15 or more	23	62.2%	21	55.3%
Grade level of students in caseload				
Elementary School (K-5)	13	35.1%	16	42.1%
Middle School (6–8)	16	43.2%	19	50.0%
High School (9–12)	20	54.1%	23	60.5%
% Students receiving special education services in caseload				
None	0	0.0%	1	2.6%
1–25%	12	32.4%	9	23.7%
26–50%	11	29.7%	14	36.8%
51–75%	6	16.2%	6	15.8%
76–100%	8	21.6%	8	21.1%
% English Language Learners in Caseload				
None	5	13.5%	7	18.4%
1–25%	21	56.8%	23	60.5%
26–50%	9	24.3%	3	7.9%
51–75%	2	5.4%	4	10.5%
76–100%	0	0.0%	1	2.6%

### Procedure

#### Recruitment

Participants were recruited via numerous professional networks, listservs, and social media (e.g., statewide organizations of school-based mental health practitioners in Illinois and North and South Carolina; a national newsletter devoted to school mental health; Twitter). Inclusion criteria were minimal to enhance generalizability and only included the requirements that (1) participants routinely provided individual-level mental health interventions or therapy and (2) spent ≥50% of their time providing services in schools. This was done to help ensure the representativeness of the sample compared to the clinicians who would ultimately access the training and consultation supports when later disseminated at a large scale. The study team conducted informed consent meetings via phone with prospective participants during which they were provided with a description of the study—including the conditions to which they could potentially be randomized—and its benefits. Participants provided verbal consent consistent with procedures approved by the University of Washington institutional review board. Recruitment lasted for a period of approximately 6 weeks to achieve the desired sample size.

#### Randomization

All participants were randomly assigned to either a BOLT+PTC condition or a service as usual control condition (see below) using the list randomizer function available at Random.org. For clinicians in the BOLT+PTC condition (*n* = 37), we conducted a scheduling survey to identify preferred times for consultation calls and then randomized the resulting consultation time blocks to our different consultation duration conditions. Using this approach, clinicians were placed into 2 (*n* = 14), 4 (*n* = 10), or 8 (*n* = 13) weeks of consultation. Clinicians in the control condition only completed study measures while continuing to provide services as usual. Blinding was not possible as clinicians were aware of their training and consultation obligations. See Additional File 1 for the study CONSORT diagram.

#### Data collection

All data were collected via online surveys and self-reported by participants. After enrolling in the study, all participants completed pre-training measures of their demographic, professional, and caseload characteristics and MBC knowledge, skill, attitudes, and use. These measures were collected weekly for 32 weeks following study enrollment. Participants received incentives in the form of $300 in the services as usual condition and $500 in the online training and consultation conditions for data collection activities (differences were due to differences in data collection burden).

#### Training and consultation

The online training and post-training consultation strategies were developed via an iterative human-centered design process intended to ensure their efficiency and contextual fit [67, 68].

##### Online training

After completing pre-training measures, participants assigned to any BOLT+PTC condition were asked to complete the online training within 2 weeks. Training included a series of interactive modules addressing the following content: (1) utility of MBC in school mental health; (2) administration and interpretation of measures; (3) delivery of collaborative feedback; (4) treatment goal-setting and prioritization of monitoring targets; (5) selecting and using standardized assessments; (6) selecting and using individualized assessments; (7) assessment-driven clinical decision-making; and (8) strategies to support continued use. The interactive online training modules are accompanied by a variety of support materials (e.g., tools to help integrate MBC into clinicians’ workflow; job-aids for introducing assessments and providing feedback on assessment results; a commonly used youth measures reference guide) via an online learning management system.

##### Consultation

The consultation model consisted of (1) 1-h small group (3–5 clinicians) live consultation sessions led by an expert MBC consultant which occurred every other week during the consultation period (e.g., clinicians in the 2-week condition had a single consultation call) and (2) asynchronous, message board discussions (hosted on the learning management system and moderated by the same consultant). Clinicians were asked to post a specified homework assignment on the message board (a) prior to their first call and (b) following each call during their assigned consultation period. Regardless of the consultation dosage, live consultation calls followed a standard sequence: (a) introduction and orientation to the session; (b) brief case presentations including MBC strategies used; (c) group discussion of appropriate next MBC steps for the case, including discussion of alternative therapeutic approaches/ strategies if MBC indicates that a change in treatment target or intervention strategy is needed; (d) expert consultant recommendations (as appropriate); and (e) wrap up, homework assignments, and additional resources. Asynchronous message board discussion provided a central location where clinicians reported on their experiences completing homework. Across the 2-, 4-, and 8-week groups, participating clinicians posted an average of 1.6 (range 0–3), 3.9 (range 0–6), and 6.2 (range 1–16) times, respectively.

#### Services as usual

Typical education sector mental health services tend to include a diverse array of assessment strategies that may include some inconsistent use of formal assessment and monitoring measures [56, 62]. Clinicians in this condition only completed study assessments.

### Measures

#### Clinician demographic, professional, and caseload characteristics

Clinician demographic, professional, and caseload characteristics were collected using a self-reported questionnaire developed by the study team, informed by those used in prior school-based implementation research (e.g., [69]). Participants completed this questionnaire upon study enrollment.

#### MBC Knowledge Questionnaire (MBCKQ)

Modeled on the Knowledge of Evidence-Based Services Questionnaire [70], the MBCKQ was designed to assess factual and procedural knowledge about MBC. The 28-item, multiple-choice MBCKQ was iteratively developed based on the key content and learning objectives of the MBC training modules and administered at baseline and 2, 4, 6, 8, 10, 16, 20, 24, 28, and 32 weeks.

#### MBC skill

Clinicians responded to 10 Likert-style items assessing MBC skills including selection and administration of measures, progress monitoring, treatment integration/modification based on the results, and feedback to clients. Responses range from 1 (“Minimal”) to 5 (“Advanced”). This scale has previously demonstrated good internal consistency (*α* = .85) when used with school-based clinicians [56]. In the current sample, *α* = .90. It was also administered at baseline and subsequently at 2, 4, 6, 8, 10, 16, 20, 24, 28, and 32 weeks.

#### MBC attitudes

The Monitoring and Feedback Attitudes Scale (MFA) [71] was used to assess clinician attitudes toward ongoing assessment of mental health problems and the provision of client feedback (e.g., “negative feedback to clients would decrease motivation/engagement in treatment”). Responses range from 1 (“Strongly Disagree”) to 5 (“Strongly Agree”). The MFA has two subscales: (1) Benefit (i.e., facilitating collaboration with clients) and (2) Harm (i.e., harmful for therapeutic alliance, misuse by administrators). In the current sample, the MFA subscales demonstrated strong internal reliability (*α* = .91 and .88, respectively) and was administered at baseline and 2, 4, 6, 8, 10, 16, 20, 24, 28, and 32 weeks.

#### MBC practices

Clinician self-reported use of MBC practices was measured by their completion of the Current Assessment Practice Evaluation – Revised (CAPER) [52], a measure of MBC penetration. The CAPER is a 7-item self-report instrument that allows clinicians to self-report their use of assessments in their clinical practice during the previous month and previous week. Clinicians indicate the percentage of their caseload with whom they have engaged in each of the seven assessment activities. Response options for each activity are on a 4-point scale (1 = “None [0%],” 2 = “Some [1-39%],” 3 = “Half [40-60%],” 4 = “Most [61-100%]”). The CAPER has three subscales, which are (1) Standardized assessments (e.g., % of case load administered standardized assessment during the last week); (2) Individualized assessments (e.g., % of caseload systematically tracked individualized outcomes last week); and, (3) Treatment modification (e.g., % of clients whose overall treatment plan altered based on assessment data during the last week). Previous versions have been found to be sensitive to training [43]. The CAPER demonstrated good internal consistency across its three subscales in the current study (*α* = .85, .94, .87). Clinicians completed the CAPER every week for 32 weeks, including baseline; total CAPER scores for each subscale were used as implementation outcomes.

### Analyses

The main goal of the current study was to test whether the BOLT+PTC strategies led to improvements in MBC implementation mechanisms and outcomes, relative to no-training services as usual condition. Specific MBC practices we measured using the CAPER were standardized and individualized assessment use and treatment modification informed by assessment data collected. Our second goal was to test whether the *dose* of PTC was differentially related to implementation outcomes (i.e., MBC practices). Finally, we tested the main effects of training and consultation dose on consultation mechanisms (i.e., MBC knowledge, attitudes, and skill).

We used R [72] for all analyses and tested our main hypotheses using multilevel models (MLM) with the statistical package ‘nlme’ [73]. MLMs allow for the analysis of clustered data, such as multiple observations of clinicians collected over time, and allows for missing data at the observation level. MLMs allowed us to simultaneously estimate all key hypothesis tests for each outcome in a single model: intervention effects on the levels of the outcome following training, intervention effects on the rate of change in the outcome over 32 weeks during and following training, and how much did PTC dose matter. These models are more appropriate than traditional ANCOVA models because they allow the integration of multiple time points into analyses while having more flexible assumptions (e.g., modeling *both* how clinicians changed over time in implementation outcomes as well as modeling differences across groups at specific time points).

We centered time such that the intercept reflected the end of consultation, *regardless of consultation dosage*. For instance, for those with 2 weeks of PTC, the intercept was centered at week 4 and for those with 4 weeks of PTC, the intercept was centered at week 6. This means that the main effects of intervention reflect differences across groups in levels of the outcomes at the end of consultation, or after the first 2 weeks of observations for the no-consultation control group.

To properly estimate the shape of change over time, we compared polynomials (quadratic and cubic effects) to a piecewise model, which estimated separate models of change during the consultation period and change in the post-consultation period. Models were chosen based on fit; we selected a given model as “better” fitting when all indices (e.g., BIC, AIC and -2LL test) agreed to avoid capitalizing on chance. These model fit indices allow comparison of the relative goodness of fit of different model specifications, balancing fit against parsimony [74]. Because we had a relatively small sample size of clinicians, we only estimated random intercepts and linear slopes; estimating random quadratic slopes (which estimate individual differences in quadratic change over time) produced model convergence problems.

We used 3 orthogonal contrast codes to compare the effects of intervention across conditions. The first compared the effects of BOLT+PTC to control. The second compared the effects of receiving PTC for 4 or 8 weeks to receiving PTC for 2 weeks, and the final contrast code compared receiving 4 vs. 8 weeks of PTC.

#### Power

The sample size balanced feasibility (e.g., the number of clinicians we expected to be able to recruit during the project period) against sensitivity of the data analytic models to detect the expected effects. We used Monte Carlo simulations in MPlus 6.0 to provide power estimates for the main effects models used in the current study. In a Monte Carlo simulation, data are simulated for a population based on estimated parameter values, multiple samples are drawn from that population, and a model is estimated for each one. Parameter values and standard errors are averaged over the samples, and power is derived based on the proportion of replications in which the null hypothesis is correctly rejected. We estimated power across 1000 replications for *n* = 75 participants measured at 32 time points. In general, our power analyses suggested that the current study had sufficient power (1 − *b* .80, *a* = .05) to detect small (*b* = .12) effects at Level 1 of the time-varying training and consultation effects across all models. On the other hand, power to detect between group differences, especially within the different consultation groups, was more limited. For example, we were powered (1 − *b* .80, *a* = .05) only to detect large (*d* = .98) differences between different consultation groups, and moderate (*d* = .58) differences between PTC and training only groups.

## Results

### Descriptive statistics

Table 1 presents overall descriptive statistics for the main sample for the current study. Table 2 presents means and SDs for implementation outcomes and mechanisms across time.

**Table 2:** Implementation outcomes and mechanisms across time

Measure	Baseline				Week 8				Week 16				Week 24				Week 32			
	BOLT		Control		BOLT		Control		BOLT		Control		BOLT		Control		BOLT		Control	
	M	SD	M	SD	M	SD	M	SD	M	SD	M	SD	M	SD	M	SD	M	SD	M	SD
**Outcomes (MBC practices)**																				
Standardized assessment	2.135	0.928	2.128	0.923	1.889	0.809	1.838	0.868	2.152	0.958	1.796	0.837	2.185	0.867	1.952	0.915	2.028	0.810	1.685	0.813
Individualized assessment	2.000	1.006	2.385	1.085	2.236	1.072	1.914	0.959	2.515	1.189	1.931	0.880	2.431	1.015	1.986	0.887	2.514	1.032	1.959	0.931
Treatment modification	1.571	0.686	1.897	0.829	1.625	0.659	1.729	0.817	1.652	0.667	1.722	0.815	1.861	0.780	1.743	0.701	1.833	0.665	1.662	0.708
**Mechanisms**																				
MBC knowledge	0.750	0.085	0.725	0.115	0.838	0.101	0.761	0.140	0.821	0.105	0.755	0.142	0.827	0.107	0.750	0.146	0.831	0.110	0.755	0.163
MBC attitudes																				
Benefit	4.343	0.473	4.305	0.448	4.358	0.493	4.137	0.403	4.448	0.438	4.236	0.393	4.425	0.462	4.080	0.668	4.417	0.438	4.276	0.451
Harm	2.101	0.605	2.231	0.779	2.069	0.750	2.257	0.789	2.061	0.628	2.264	0.763	2.049	0.649	2.229	0.651	2.076	0.746	2.169	0.651
MBC skill	3.399	0.797	3.494	0.767	3.660	0.705	3.525	0.654	3.924	0.573	3.476	0.765	3.743	0.648	3.339	0.729	3.903	0.700	3.625	0.641

### Main effects on implementation outcomes

First, we examined whether receiving BOLT+PTC produced changes in MBC practices during the post-consultation period (weeks 8–32), relative to the control group, and without accounting for change over time. Generally, mean differences were moderate for use of standardized assessment (Cohen’s *d* = .37, *p* = .11) and individualized assessment (Cohen’s *d* = .49, *p* = .04), but close to zero for treatment modification (Cohen’s *d* = .05, *p* = .84). However, it is important to note that these main effect estimates were non-significant or trending towards non-significance.

### Change over time in outcomes

**Fig. 1:**
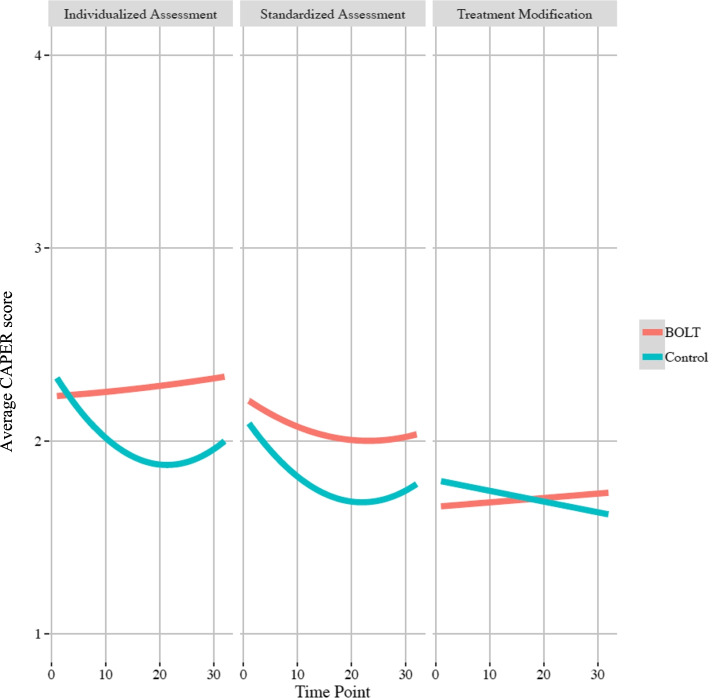
Main effects of BOLT on main outcomes over time

Next, we modeled change over time in the main outcomes, MBC practices. For standardized assessment, a quadratic term exhibited best fit to the data indicating a curvilinear pattern of change (see Fig. 1), but a linear only model fit the data for individualized assessment and treatment modification.

### Main effects over time

We tested whether receiving BOLT+PTC led to post-training changes in MBC practices over time. Table 3 presents these findings. Results indicated relatively similar findings across outcomes, in that those clinicians who received training and any consultation exhibited better outcomes over time, while those in the control group exhibited declines in MBC practices over time, with those declines generally slowing with time. Figure 1 also illustrates the effects of BOLT+PTC over time on MBC practices. Specifically, for both *standardized assessment* (*b* = .02, *p* = <.01) and *individualized assessment* (*b* = .04, *p* < .001), there was a significant intervention effect on change over time, such that participants in the BOLT+PTC condition exhibited less decline relative to those in the control condition. While clinicians in BOLT+PTC continued to report higher levels of standardized and individualized assessments, control group participants reported declines over time. On the other hand, we observed no effects of BOLT+PTC on *treatment modification*.

**Table 3:** Main effects of BOLT+PTC on outcomes over time

	*Standardized assessment*			*Individualized assessment*			*Treatment modification*		
	***b***	S.E.	***p***	***b***	S.E.	***p***	***b***	S.E.	***p***
Intercept	2.05	0.12		2.28	0.13	<0.001	1.79	0.09	
Week (Linear)	**−0.04**	0.01	<0.001	**−0.04**	0.01	<0.001	**−**0.01	0.00	0.07
Week (Quadratic)	**0.0094**	0.0002	<0.001	**0.0011**	0.0003	<0.001			
BOLT+PTC vs. Control	0.06	0.16	0.71	**−**0.04	0.18	0.83	**−**0.11	0.12	0.36
Week (Linear) × BOLT+PTC vs. Control	**0.02**	0.01	<0.01	**0.05**	0.01	<0.001	0.01	0.00	0.08
Week (Quadratic) × BOLT+PTC vs. Control	**−**0.0005	0.0002	0.09	**−0.0011**	0.00036	<0.001			

Bolded coefficients are significant at *p* < .05. Coefficients are rounded to the nearest non-zero decimal place

### Effects of PTC dose on main outcomes over time

We next explored whether PTC dose influenced outcomes, by replicating the analyses above with additional contrast codes for different PTC doses. Across models, there was little evidence of a consistent pattern of findings. Although some effects reached the traditional significance level, there was not consistent or strong evidence that any one PTC dose condition had a stronger impact on outcomes than any other. Because of the small sample size of the individual training groups, and the inconsistent effects, we refrain from interpreting them as evidence of a clear dose effect of PTC. See Table 4.

**Table 4.** Effects of consultation dose on BOLT outcomes over time

	*Standardized assessment*			*Individualized assessment*			*Treatment modification*		
	*b*	S.E.	*p*	*b*	S.E.	*p*	*b*	S.E.	*p*
Standardized assessment intercept	2.08	0.09		2.24	0.10		1.70	0.07	
Week (Linear)	**−0.02**	0.00	<0.001	**−**0.01	0.01	0.23	0.00	0.00	0.78
Week (Quadratic)	**0.0006**	0.0002	<0.01	0.0002	0.0002	0.24	–		
BOLT vs. Control	0.03	0.12	0.823	**−**0.04	0.13	0.77	**−**0.09	0.09	0.33
2-week PTC vs. 4/8-week PTC	0.02	0.15	0.92	**−**0.29	0.17	0.01	**−**0.05	0.12	0.66
4 vs. 8-week PTC	0.00	0.14	0.98	0.06	0.15	0.71	0.03	0.11	0.74
Week (Linear) × BOLT vs. Control	**0.018**	0.007	<0.01	**0.04**	0.008	<0.001	0.006	0.003	0.06
Week (Linear) × 2-week PTC vs. 4/8-week PTC	**−0.016**	0.008	0.04	0.0048	0.009	0.61	**−**0.003	0.004	0.38
Week (Linear) × 4 vs. 8-week PTC	0.003	0.006	0.65	**−0.0016**	0.007	0.03	**−0.007**	0.004	0.05
Week (Quadratic) × BOLT vs. Control	**−**0.0004	0.0002	0.12	**−0.0008**	0.0003	<0.01	–		
Week (Quadratic) × 2-week PTC vs. 4/8-week PTC	**0.0006**	0.0003	0.04	**−**0.00004	0.0004	0.93	–		
Week (Quadratic) × 4 vs. 8-week PTC	0.0002	0.0003	0.60	0.0006	0.0003	0.06	–		

Bolded coefficients are significant at *p* < .05. Coefficients are rounded to the nearest non-zero decimal place

### Effects on implementation mechanisms

Next, we tested whether BOLT+PTC was associated with change over time in the theoretical implementation mechanisms (MBC knowledge, attitudes, and skill). We used a similar approach to the analyses above, using multilevel models with time centered at the end of consultation for each group. At the end of consultation, participants in the BOLT+PTC conditions reported higher levels of MBC knowledge (*b* = .06, *p* = .019), but not higher levels of MBC skill (*b* = .13, *p* = .33), or more positive MBC attitudes including perceived benefit (*b* = .16, *p* = .06) or perceived harm (*b* = −.12, *p* = .34) compared to control group participants. Participants in the BOLT+PTC condition exhibited larger increases across the consultation period in MBC skills (*b* = .03, *p* <.001), although these increases slowed over time (*b* = −0.00091, *p* = <.001), and there were ultimately no differences at the end of consultation. We observed no other effects of BOLT+PTC on rates of change over time.

We also explored whether PTC dose was associated with differential outcomes for theorized mechanisms (skill, attitudes, and knowledge), by replicating the analyses above with additional contrast codes for different PTC doses. Across all outcomes, there was no evidence that PTC dose was associated with differences in mechanisms.

## Discussion

This study tested the impact of an efficient, brief online training and post-training consultation program for MBC with school-based mental health clinicians. In addition to examining the effects of BOLT+PTC, it was the first study to experimentally manipulate PTC dose to evaluate the amount of support that may be needed to promote effective implementation. Consistent with current implementation research design guidelines to advance the precision of implementation science results, we examined the impact of BOLT and various dosages of BOLT+PTC on implementation outcomes (e.g., MBC practices) as well as implementation mechanisms (i.e., MBC knowledge, attitudes and skill).

Beyond examinations of dose, the precision of implementation science is increased by better specification of implementation outcomes. In the current project, effects were apparent over time for the impact of BOLT+PTC on both standardized and individualized assessment use, but not for treatment modification, even though each of these was an explicit focus of the training and consultation. Consistent with this, the developers of the CAPER have reported the lowest mean ratings for the treatment modification subscale [52]. Prior findings also have found less change—and less sustainment of changes—in treatment modifications following training and consultation in MBC practices [43]. It may be the case that changing one’s practice to collect standardized or individualized measures is more straightforward than using those measures to inform treatment modifications. Indeed, determining changes to treatment plans is often more complicated than instrument administration alone. Studies have pointed to the importance of ensuring clinical decision support during MBC implementation [75], which some measurement feedback systems and ongoing clinical consultation can provide. Although MBC has been associated with improved outcomes among a wide variety of presenting concerns and treatment modalities [44, 45], it is possible that using MBC for treatment planning is more ambiguous when implemented without a specific evidence-based intervention. The current sample of clinicians delivered MBC-enhanced “usual care” mental health services in schools. Treatment modification may be easier if MBC is implemented alongside a broader practice change initiative, such as those that include transdiagnostic or common elements of evidence-based practices [76–78], or even clear and consistent specification of usual care treatment elements [79].

Notably, these clinicians also delivered usual care services in the education sector, where there is limited research on evidence-based practice implementation [61]. Given the importance of better understanding setting-specific rates of change, deterioration, outcomes, and treatment interventions that may influence MBC implementation and effectiveness [80], additional research examining how MBC practices can more effectively inform treatment modifications in school mental health treatment—and beyond—is greatly needed.

Regarding implementation mechanisms, one of the three theorized mechanisms was impacted for the BOLT+PCT group, relative to controls. Specifically, MBC knowledge was higher immediately following consultation. In contrast, EBP attitudes have been inconsistently associated with implementation outcomes in prior research across service sectors and interventions [81, 82]. Given that some implementation strategies have been found to successfully shift practitioner attitudes in the education sector (e.g., Beliefs and Attitudes for Successful Implementation in Schools [BASIS] [83, 84]), there might be utility in focusing more explicitly on that mechanism to enhance the utility of BOLT approach.

Research is increasingly investigating “how low can you go” with regard to implementation processes and pursuing pragmatic and cost-effective implementation supports [37, 85, 86]. Related, our findings regarding consultation dose were not as anticipated. Specifically, we observed very small differences among the different PTC groups (2, 4, or 8 weeks), indicating that while post-training consultation is critical, higher doses may not have improved its impact in this sample. However, many questions remain surrounding optimal consultation dosage, and replication with larger samples of clinicians is needed. Aside from aforementioned between-group power limitations, there are several possible explanations for our findings. First, it may be that MBC practices are simpler, relative to evidence-based treatment protocols which often use training and PTC to support clinicians’ adoption and ongoing implementation with fidelity. Therefore, perhaps less consultation is more likely to be adequate for MBC than for manualized, evidence-based interventions. Indeed, it has been suggested that less complex interventions may need fewer implementation supports to be successfully adopted [87]. On the other hand, our findings that standardized and individualized assessment administration changed more over time as compared to treatment modifications suggests that treatment modification could be a more complex or challenging practice to change than just collecting new measures. Additionally, changes in treatment modification may be less frequently indicated than other changes in MBC practice, as they are often dependent on the results of the assessment (e.g., in cases of nonresponse or deterioration). It is also possible that none of the consultation conditions were sufficient to effect this change or to support clinicians in determining what other intervention changes were indicated when measurement suggests a lack of progress. Such a “ceiling effect” for our specific set of training and PTC strategies could necessitate the incorporation of some additional techniques, such as the BASIS strategy noted earlier. In BOLT+PTC, treatment modifications in response to assessment data were discussed more generally to enhance usual care, rather than in adherence to a particular set of intervention practices or expectation to deliver a manualized intervention. Future work should explore whether the brief model could be augmented to focus more explicitly on understanding when or how to adjust care. In this way and others, MBC—and MBC-facilitated treatment more generally—may continue to be a practice that is “simple, but not easy.”

## Limitations

Findings of the current study must be interpreted within the context of several limitations. First, we were unable to include observational measures of MBC practices in this initial study. Although self-reported clinical practices are typical in implementation research for practicality and resource constraints particularly of pilot trials, observational measures would have been more robust and are a recommended future direction for related work. A review of clinical records was similarly infeasible due to the remote nature of this study and the diverse systems in which participants worked. Second, the frequency with which MBC practices were assessed may have had an impact on clinicians’ MBC behaviors, although this assessment approach was consistent across conditions. Third, it is not entirely clear why some deterioration of MBC practices was observed over time in our sample. It may be that the baseline ratings of MBC practices were inflated and that repeated assessments produced ratings that were more reflective of clinicians’ behaviors. The pattern of results observed from weeks 8 to 32 (Table 2) provides some support for this possibility. Fourth, based on previously described research documenting the limitations of workshop training alone, we opted not to include a training-only condition to examine the differential effects of training compared to training plus consultation. Fifth, collection of student clinical outcomes was outside the scope of this study due to its pilot nature and our explicit focus on the most proximal implementation mechanisms and outcomes linked to the implementation supports provided. Sixth, although we examined main effects on hypothesized implementation mechanisms, we did not conduct formal tests of mediation as they were beyond the scope of the current paper. Finally, as indicated above, the sample sizes of each PTC dosage condition limited the conclusions that could be drawn about the differential effects of a greater number of weeks of consultation.

## Conclusion and future directions

Results from this study indicate that efficient training and consultation supports can produce meaningful practice change surrounding the administration of assessments. However, better understanding the optimal dosage of consultation when paired with high-quality, active training requires further investigation, particularly for MBC delivered in schools. This is in line with recent evidence that even a condition without any post-training consultation—but with other potentially low-cost (but high quality) supports like handouts, video tutorials, and access to online supports—was able to yield improvements in some aspects of fidelity, including those that might be most important for patient outcomes [8]. This points to the necessity of continuing to identify and test the mechanisms engaged by various implementation strategies, especially training and consultation processes, to determine the most parsimonious and efficient approaches. Certainly, the incremental impact of consultation relative to alternative or adjunctive post-training support strategies, such as measurement feedback systems [88, 89], should be further explored as research on successful MBC implementation strategies unfolds.

## Supplementary Information

**Additional file 1.** CONSORT Flow Diagram.

## Data Availability

The datasets generated during and/or analyzed during the current study are not publicly available but are available from the corresponding author on reasonable request.

## Abbreviations

BOLT: Brief online training
CAPER: Current Assessment Practice Evaluation – Revised
MBC: Measurement-based care
MBCKQ: Measurement-Based Care Knowledge Questionnaire
MFA: Monitoring and Feedback Attitudes Scale
MLM: Multilevel model
PTC: Post-training consultation
