# Supplementary material for: How low can you go? Examining the effects of brief online training and post-training consultation dose on implementation mechanisms and outcomes for measurement-based care
Source: Implement Sci Commun. 2022 Jul 22;3:79. doi: 10.1186/s43058-022-00325-y (PMC9306246; doi:10.1186/s43058-022-00325-y)
Supplement: Supplementary file 1 — Additional file 1. CONSORT Flow Diagram. [file 43058_2022_325_MOESM1_ESM.doc]

**CONSORT Flow Diagram**

**Allocation**

**Enrollment**

**Analysis**

**Follow-Up**

Assessed for eligibility (n= 117)

Excluded or declined (n= 35)

Allocated to BOLT+PTC (n=42)

Allocated to Service as Usual/Control (n=40)

♦ Received allocated intervention (n=40)

♦ Did not receive allocated intervention (n= 0)

Randomized (n= 82)

Analysed (n=37)
♦ Excluded from analysis (n=0)

Lost to follow-up (n=3)

- Unknown reason/unresponsive

Discontinued intervention (n= 0)

Analysed (n=38)
♦ Excluded from analysis (n=0)

Randomized (n= 42)

2 Week PTC (n=14)

4 Week PTC (n=14)

8 Week PTC (n=14)

♦ Received allocated intervention (n=10)

♦ Did not receive (n= 4)

♦ Received allocated intervention (n=13)

♦ Did not receive (n= 1)

♦ Received allocated intervention (n=14)

♦ Did not receive (n= 0)

Lost to follow-up (n=1)

- Unknown reason/unresponsive

Discontinued intervention (n= 0)
